# MRI Characterization of Non-traumatic Intracerebral Hemorrhage in Young Adults

**DOI:** 10.3389/fneur.2020.558680

**Published:** 2020-10-29

**Authors:** Mohamed Elmegiri, Riku-Jaakko Koivunen, Turgut Tatlisumak, Jukka Putaala, Juha Martola

**Affiliations:** ^1^Department of Radiology, Medical Imaging Center, University of Helsinki and Helsinki University Hospital, Helsinki, Finland; ^2^Department of Anesthesiology and Intensive Care Medicine, University of Helsinki and Helsinki University Hospital, Helsinki, Finland; ^3^Department of Clinical Neurosciences, Institute of Neurosciences and Physiology, Sahlgrenska Academy at University of Gothenburg, Gothenburg, Sweden; ^4^Department of Neurology, Sahlgrenska University Hospital, Gothenburg, Sweden; ^5^Department of Neurology, University of Helsinki and Helsinki University Hospital, Helsinki, Finland

**Keywords:** spontaneous intracerebral hemorrhage, young adults, magnetic resonance imaging, structural cause, cavernoma

## Abstract

**Background and Purpose:** Non-traumatic intracerebral hemorrhage (ICH) in younger population is a relatively rare event but is associated with considerable mortality and poor functional outcome. Imaging plays a crucial role in determining the underlying cause and guide treatment of ICH. In up to 41% of patients in prior studies, the underlying cause remained elusive. However, the usage of MRI as part of diagnostic work-up was scanty. We aimed to analyze MRI findings of ICH in younger patients and assess specificity and sensitivity of MRI in detecting structural or local underlying causes of ICH.

**Methods:** We included patients aged 15–49 years with first-ever ICH identified from a prospective hospital discharge registry, 2000–2010. All study patients underwent MRI within 3 months of ICH. Imaging data was analyzed by a senior neuroradiologist blinded to final clinical diagnosis. We calculated the diagnostic accuracy of MRI in detecting structural/local underlying causes.

**Results:** Of our 116 patients (median age, 39; 67% males), structural/local causes were the leading causes of ICH (50.0%), and of these, bleeding cavernomas (23.3%) were the most frequent followed by arteriovenous malformations (12.9%), cerebral venous thrombosis (CVT) (7.8%), brain tumors (5.2%), and moyamoya disease (0.9%). Lobar location of ICH was more prevalent in younger patients. MRI was highly sensitive (90.0%; 95% confidence interval, 79.5–96.2%) for detection of structural/local causes compared with angiographic imaging (55.6%; 95% CI, 40.0–70.4%), while MRI was less specific (87.3%; 95% CI, 75.5–94.7%) for structural/local causes, compared with angiographic imaging (97.4%; 95% CI, 86.5–99.9%).

**Conclusion:** MRI was highly sensitive for the detection of structural and local causes underlying ICH in young adults. Thus, MRI should be considered in the diagnostic work-up of all young ICH patients to enable targeted secondary prevention.

## Introduction

Non-traumatic intracerebral hemorrhage (ICH) in younger population, aged between 18 and 50 years, is a relatively rare event but is associated with a substantial mortality and poor long-term functional outcome (1). According to a recent study comparing functional outcome between main subtypes of stroke in the young, of all young stroke patients who had a poor functional outcome, 49.3% were associated with ICH, while 36.5% were associated with ischemic stroke, and 16.8% with transient ischemic attack (2).

Determining the likely etiology underlying ICH is of utmost importance in order to reduce the risk of rebleeding, targeting ancillary testing, and counseling patients. Nevertheless, etiological diagnosis in young patients with ICH is often challenging. In a number of studies that have been conducted to determine the common underlying causes of ICH in young adults, up to 41% (mean 22%) was assigned to have an undetermined cause (1). The most common reported causes of ICH in young patients were hypertensive microangiopathy (25%) and vascular malformations (25%), while cavernous hemangiomas were the most common of vascular malformations. Cerebral venous thrombosis (CVT) and illicit drug abuse were less common causes (1, 3). Inflammatory or non-inflammatory vasculopathies, such as vasculitis, posterior reversible encephalopathy syndrome (PRES), reversible cerebral vasoconstriction syndrome (RCVS), and neoplasms were rare causes of ICH in the young.

Imaging has a crucial role in determining the underlying etiology of ICH. The most recent American Stroke Association (ASA) guidelines recommend emergent CT as the initial imaging to distinguish ischemic stroke from intracranial hemorrhage (4). However, ICH in younger patients almost invariably requires further investigations. Contrast-enhanced CT or MR with angiography and/or venography, as well as digital subtraction angiography (DSA) may be helpful in determining the underlying abnormalities, such as vascular malformations, neoplasms, and CVT.

According to a 2018 review, which included 13 studies investigating ICH in young adults with a total of 2,121 patients (1), only 3 studies reported the performance of brain MRI during the initial imaging workup for clinically suspicious ICH young adults, with an average proportion of 22.2% (3, 5, 6). Four studies did not report the proportion of patients undergoing brain MRI (7–10), while the rest of the studies reported no usage of MRI (11–16). Up to 41% (mean 22%) of patients were diagnosed as having no detectable underlying cause (1, 15). Notably, the detection of underlying structural causes was higher in studies, which included MRI in the diagnostic work-up imaging in addition to DSA, compared with those studies with only DSA being performed without any reported MRI imaging.

Due to the paucity of data regarding the utility of MRI of ICH in young adults, we aimed to provide a better characterization of MRI features of ICH in young adults and to assess additive yield of MRI besides other imaging modalities.

## Materials and Methods

Helsinki Medical Imaging Center at the Helsinki University Hospital (HUH) is the sole provider of constantly 24/7 imaging service in the Helsinki metropolitan area, with a catchment area of around 1.5 million people. Departments of Neurology and Neurosurgery at HUS form a designated stroke center, receiving referrals from the other district hospitals throughout the Helsinki metropolitan area. Virtually all young patients with suspected acute stroke are thus referred to HUH. All patients are prospectively recorded into hospital electronic discharge database. Clinical and imaging data are stored electronically with all information about the clinical process including detailed clinical history, diagnostic work-up, plan of management, as well as imaging and laboratory studies. As this study was based on retrospective data, ethical approval and written consents from patients were not required for the study in accordance with the local legislation and institutional requirements. Relevant institutional permission for study conduct was obtained.

### Patient Selection

We retrospectively searched all patients from the hospital discharge database with an International Classification of Diseases, 10th Revision (ICD-10) diagnosis of ICH or structural lesion with ICH as one of the primary complications (diagnosis code of I60.8, I61, I67.3, I67.4, I67.5, I67.6, I67.7, I67.8, I67.9, I68, or I78) at age between 15 and 49 over a 10-year period from 1st January 2000 to 31st March 2010, as described earlier (17).

**Figure 1:**
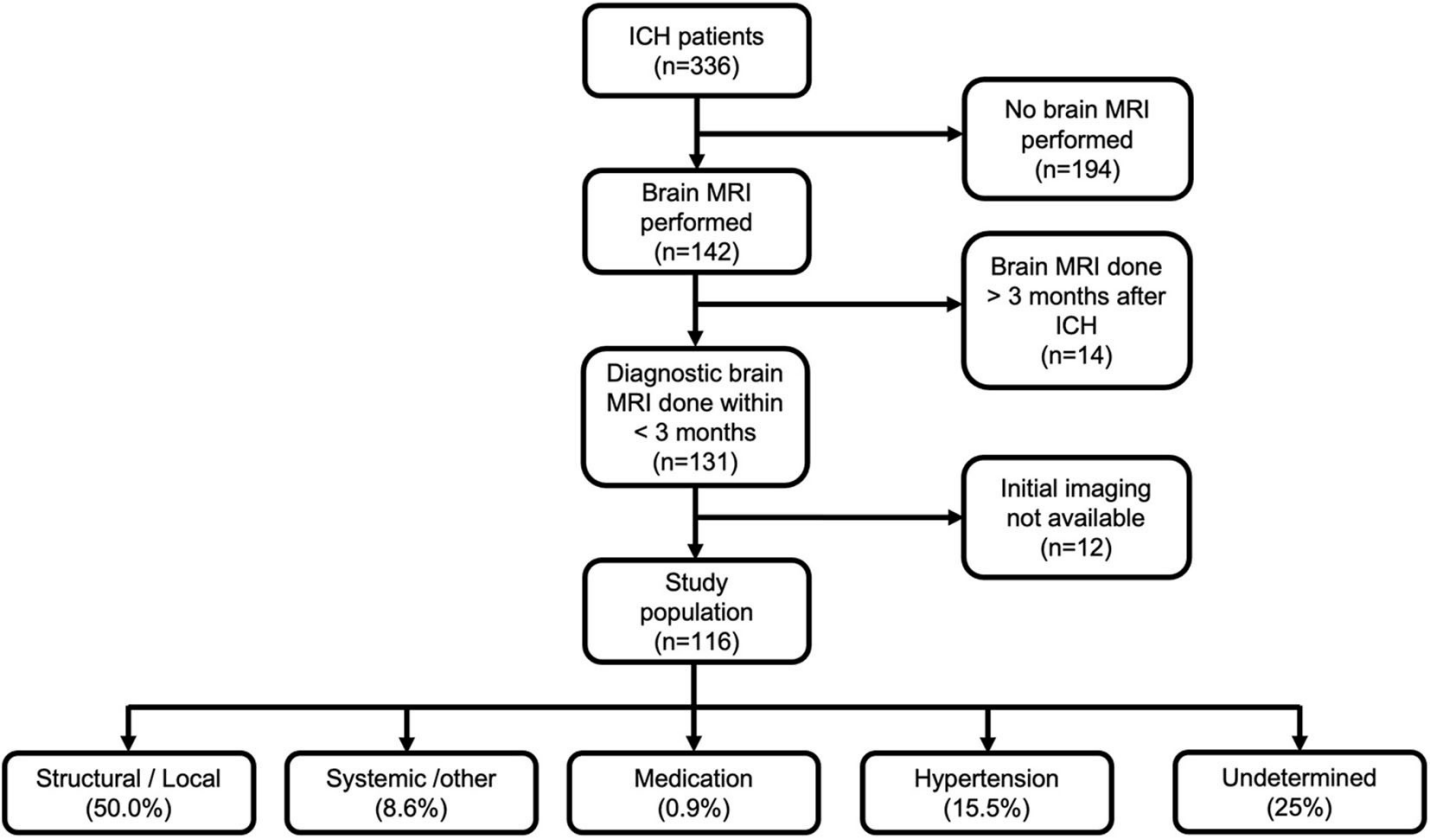
Flowchart of the study population and final etiological diagnosis according to SMASH-U classification. ICH, intracerebral hemorrhage; MRI, magnetic resonance imaging.

For the present study, we selected patients who had undergone an initial brain CT and brain MRI, followed by other ancillary imaging as deemed by the treating neurologist. In order to be included, patients had to undergo diagnostic brain MRI within 3 months of the date of first-ever ICH [median, 5 days; interquartile range (IQR), 2–20]. By excluding patients with MRI obtained >3 months from the ICH, we mainly aimed to exclude the possibility of having recurrent neurovascular events that could hamper evaluation of the index bleed. We also excluded patients who were imaged initially outside the metropolitan area of Helsinki if their imaging studies could not be retrieved for evaluation from the electronic archives. Flowchart of the study population is presented in Figure 1.

### Clinical Data

Clinical data extracted from medical records included age, sex, comorbidities, date of ICH, and dates of performed diagnostic imaging. All records of the selected patients were finally reviewed by a senior consultant neurologist (J.P.), who established the final diagnosis using all clinical and imaging data. We classified ICH etiology initially according to SMASH-U classification (18), with further dichotomization into specific structural/local cause and systemic/undetermined causes Figure 1. CVT was classified as structural/local cause for the present analysis.

### Imaging Data Analysis

All brain imaging studies were initially reviewed blinded to final clinical diagnosis by a consultant senior neuroradiologist (J.M.) with >10 years of experience, using PACS system (AGFA healthcare N.V., IMPAX 6.6, Mortsel, Belgium). We recorded dates and data on initial diagnostic imaging modalities (emergent CT or MRI scan). The modality of choice for initial imaging in the youngest patients was MRI when available, while older patients underwent usually conventional initial CT scan, followed by MRI scan within 3 months of the incident ICH. Secondary imaging modalities included one or more of the following: CT angiography (CTA) and/or venography (CTV), MR angiography (MRA) and/or venography (MRV), and/or digital subtraction angiography (DSA).

For MRI scans, basic imaging sequences were obtained including T1-weighted (T1W), T2-weighted (T2W), fluid attenuation inversion recovery (FLAIR), and conventional T2^*^-gradient echo sequences. Additional sequences were obtained based on either the initial CT radiological diagnosis or the clinical findings. Those included time-of-flight (TOF) angiography, diffusion-weighted images (DWI), contrast-enhanced T1W images, or MR venography. More recent imaging included also susceptibility-weighted imaging (SWI) sequences with added sensitivity for depiction of cerebral microbleeds (CMBs) (19, 20). MRI sequences included T2^*^ or SWI in 75.0% of the patients and in 67.2% imaging included T1WI with gadolinium contrast.

We classified location, site, and shape of ICH (regular, irregular, multiple bleedings) and the presence of intraventricular hemorrhage (IVH). ICH volume was assessed using the ABC/2 formula (21). We recorded the presence of subarachnoid hemorrhage (SAH), hydrocephalus, and combined adjunct features. Furthermore, MRI studies were analyzed for the presence of CMBs in those patients whom MRI sequences included T2^*^-weighted and/or SWI sequences.

### Statistical Analysis

For continuous variables, we report means (±SD) or median (interquartile range), and for categorical variables, we report *n* (%). Normality of continuous variables was checked, and comparisons performed with either Student's *t* or Mann-Whitney *U*-test. Comparison of clinical and MRI characteristics were carried out between two age groups dichotomized at median age.

The initial diagnoses, which were made based on different imaging modalities, were compared, and accuracy assessed in suggesting the final clinicoradiological diagnosis. Sensitivity, specificity, accuracy, as well as negative and positive predictive values were subsequently calculated for brain MRI in detecting structural/local causes underlying ICH using MedCalc online statistical calculator (22). In addition, we performed two sensitivity analyses to assess, whether age of the patient (dichotomized at age median) and timing of brain MRI (≤2 vs. >2 weeks from ICH onset) affects diagnostic performance of MRI.

*P*-value of <0.05 was considered statistically significant. Statistical analysis used SPSS version 22 for Windows (IBM, Inc., Armonk, NY, USA).

## Results

### Patient Demographics and Clinical Characteristics

A total of 116 patients were included in our study, of which 67 (57.8%) were male, with median age of 39 years (IQR, 28.5–39.3). Clinical characteristics of study populations including risk factors and final clinical diagnoses appear in Table 1. The most common risk factor among study population was hypertension, which was more prevalent in the older age group (>39 years). Structural causes were the leading causes of ICH in our study, with cavernomas (23.3%) being the most common among structural causes. Hypertension (15.5%) was the second most common etiology, being most frequent in the older age group.

**Table 1:** Clinical characteristics of the study population including risk factors and final clinical diagnoses.

	**Age group**			***P-*value**
	**Total** **(*n* = 116)**	**<39 years** **(*n* = 58)**	**≥39 years** **(*n* = 58)**	
**RISK FACTORS**				
Hypertension	22 (19.0)	6 (10.3)	16 (27.6)	0.018
Type 1 diabetes	5 (4.3)	2 (3.4)	3 (5.2)	0.648
Type 2 diabetes	1 (0.9)	0 (0.0)	1 (1.7)	0.315
Hematologic disorder	2 (1.7)	2 (3.4)	0 (0.0)	0.154
Previous stroke	4 (3.4)	2 (3.4)	2 (3.4)	1.000
History of malignancy	2 (1.7)	0 (0.0)	2 (3.4)	0.154
**PRECEDING MEDICATION**				
Antiplatelets	5 (4.3)	0 (0.0)	5 (8.6)	0.022
Antihypertensive	10 (8.6)	3 (5.2)	7 (12.1)	0.186
**ETIOLOGY**				0.027
Structural/local causes				
Cavernoma	27 (23.3)	17 (29.3)	10 (17.2)	
AVM	15 (12.9)	11 (19.0)	4 (6.9)	
CVT	9 (7.8)	7 (12.1)	2 (3.4)	
Brain tumor	6 (5.2)	3 (5.2)	3 (5.2)	
Moyamoya	1 (0.9)	0 (0.0)	1 (1.7)	
Medication				
Anticoagulation	1 (0.9)	0 (0.0)	1 (1.7)	
Illicit drugs	3 (2.6)	3 (5.2)	0 (0.0)	
Hypertension				
Hypertension	18 (15.5)	4 (6.9)	14 (24.1)	
Acute hypertensive crisis	1 (0.9)	0 (0.0)	1 (1.7)	
Systemic/Other				
RCVS	1 (0.9)	0 (0.0)	1 (1.7)	
Liver disease (cirrhosis)	1 (0.9)	0 (0.0)	1 (1.7)	
Vasculitis	3 (2.6)	1 (1.7)	2 (3.4)	
Other hematological disorder	1 (0.9)	1 (1.7)	0 (0.0)	
Unknown	29 (25.0)	11 (19.0)	18 (31.0)	

Data are n (%).

*AVM, arteriovenous malformation; CVT, cerebral venous thrombosis; RCVS, reversible cerebral vasoconstriction syndrome*.

### MRI Findings

**Figure 2:**
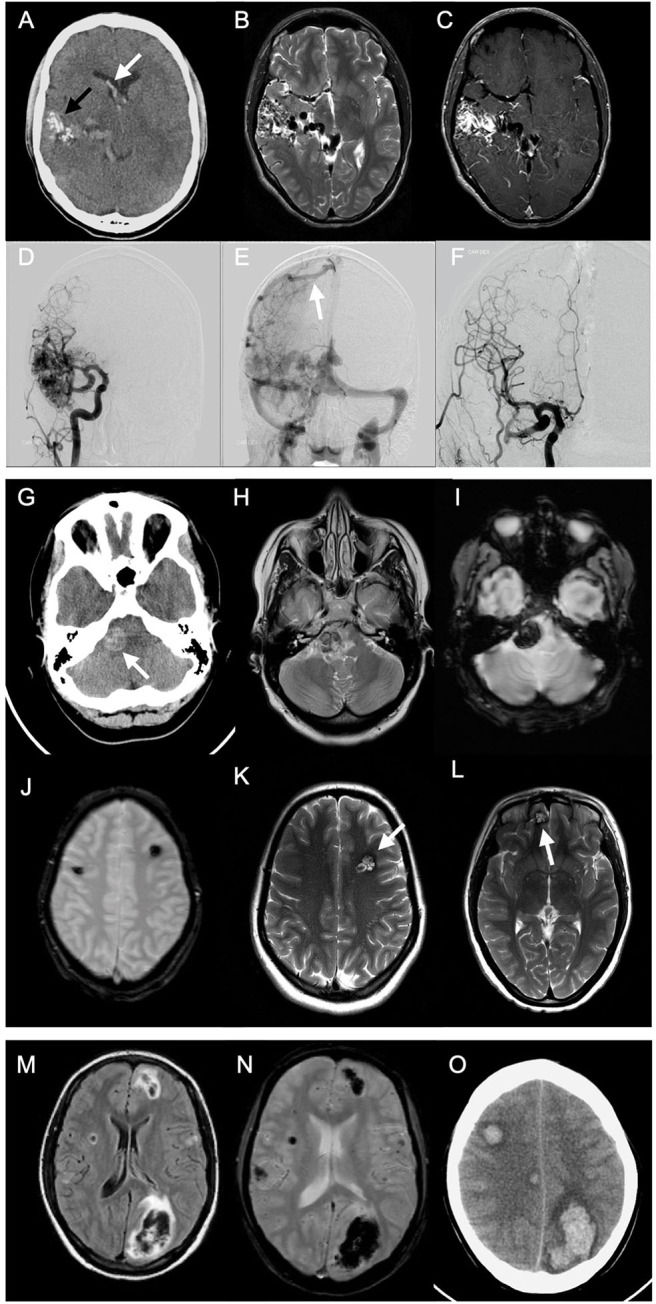
Multimodality imaging of different spontaneous intracerebral hemorrhage etiologies in young adults. *Case 1*: A 20-year-old male presented to emergency with sudden onset headache. Emergent CT was done. Axial non-enhanced CT (NECT) **(A)** shows right temporal serpentine calcifications (*black arrow*), associated with intraventricular hemorrhage (*white arrow*) and calcifications. Axial T2-weighted images (T2WI) **(B)** show classic wedge-shaped arteriovenous malformation (AVM) with flow voids representing feeding arteries and dilated draining veins. Axial T1-weighted images (T1WI) with contrast **(C)** show avid AVM nidus enhancement. Pre- **(D,E)** and postoperative **(F)** catheter angiography shows dilated middle and posterior cerebral arteries feeding AVM and dilated draining cortical vein to superior sagital sinus (*white arrow*). *Case 2*: A 24-year-old female, presented with history of headache persisting for 1 week. NECT was performed **(G)** showing hyperdense lesion at the right cerebellopontine angle. Axial T2WI **(H)** show hypointense lesion with surrounding edema. T1WI with contrast showed no contrast enhancement (not shown). T2*gradient echo (GRE) scan **(I)** shows “blooming” effect within the lesion. Another T2*GRE image **(J)** at different levels and T2WI **(K,L)** showing multiple lesions at different sites with typical “popcorn” appearance, representing multiple cavernomas. *Case 3*: A 20-year-old female with history of headache for 1 week. Emergent MRI scan was performed. Fluid-attenuated inversion recovery (FLAIR) images **(M)** show multifocal areas of hypointensities, mainly lobar in location, surrounded by hyperintense margins representing edema. T2*GRE image **(N)** shows “blooming” representing multiple acute intracerebral hemorrhages (ICHs). Two days later, follow-up NECT **(O)** showed multiple ICHs with surrounding moderate edema. Investigations revealed acute leukemia as underlying etiology.

Detailed MRI-specific features of ICH including location, site, shape, volume, presence of microbleeds, and associated complications are shown in Table 2. Lobar location of ICH was significantly more prevalent in the younger age group, while deep location (ganglionic or thalamic) was more prevalent in the older age group. There was no difference in the ICH laterality. ICH was mainly irregular in shape (67.2%) with no difference between the age groups. In 9.5% of cases, shape characterization was not applicable, for example, in cases of IVH. In 75.0% of cases, their MRI scan included either T2^*^, SWI, or both sequences for assessment of the presence of CMBs. In only 10.0% of cases, CMBs were detected with no difference between the age groups. The most common adjunct feature was IVH or SAH, with no difference between age groups. Hydrocephalus was presented either as sole complication or as a resultant complication from IVH or SAH. The ICH volumes ranged from tiny small bleedings to large massive bleedings, with median volume of 7 ml (IQR, 2.0–19.0). There was no difference between age groups in ICH volumes (*P-*value 0.595). Examples of multimodality imaging of different ICH etiologies in study participants are presented in Figure 2.

**Table 2:** Specific brain MRI findings in the entire study population and stratified according to age group.

	**Age group**			***P-*value**
	**Total** **(*n* = 116)**	**<39 years** **(*n* = 58)**	**≥39 years** **(*n* = 58)**	
**LOCATION**				0.008
Lobar	56 (48.3)	30 (51,7)	26 (44.8)	
Basal ganglia/thalamus	27 (23.3)	9 (15.5)	18 (23)	
Brain stem	15 (12.9)	4 (6.9)	11 (19)	
Cerebellum	2 (1.7)	1 (1.7)	1 (1.7)	
Intraventricular	10 (8.6)	9 (15.5)	1 (1.7)	
Mixed	6 (5.2)	5 (8.6)	1 (1.7)	
**SITE**				0.036
Right	40 (34.5)	19 (32.8)	21 (36.2)	
Left	54 (46.6)	24 (41.4)	30 (51.7)	
Bilateral	7 (6)	5 (8.6)	2 (3.4)	
Midline	5 (4.3)	1 (1.7)	4 (6.9)	
Isolated IVH	10 (8.6)	9 (15.5)	1 (1.7)	
**SHAPE**				0.099
Regular	26 (22.4)	13 (22.4)	13 (22.4)	
Irregular	78 (67.2)	35 (60.3)	43 (74.1)	
Mixed/multiple bleedings	1 (0.9)	1 (1.7)	0 (0)	
Not applicable	11 (9.5)	9 (15.5)	2 (3.4)	
**MICROBLEEDS**				0.884
No	77 (66.4)	38 (65.5)	39 (67.2)	
Yes	11 (9.5)	5 (8.6)	6 (10.3)	
Not assessable	28 (24.1)	15 (25.9)	13 (22.4)	
**COMPLICATIONS**				0.337
None	69 (59.5)	34 (58.6)	25 (60.3)	
IVH/SAH	23 (19.8)	9 (15.5)	14 (24.1)	
Hydrocephalus	5 (4.3)	4 (6.9)	1 (1.7)	
Both	19 (16.4)	11 (19)	8 (13.8)	

Data are n (%).

*IVH, intraventricular hemorrhage; SAH, subarachnoid hemorrhage*.

### Imaging Diagnoses

**Figure 3:**
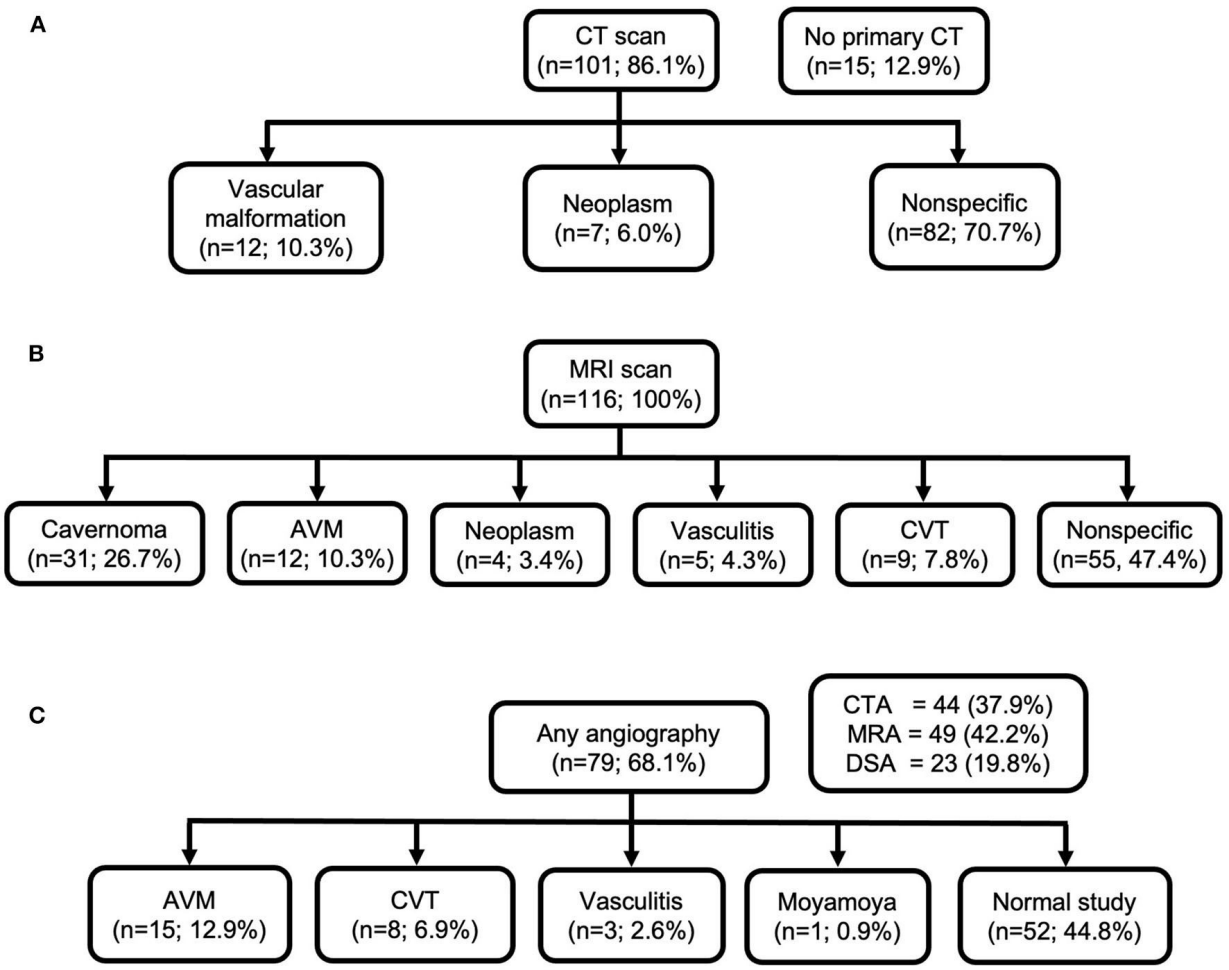
Neuroimaging performed in the study population and radiological diagnoses. **(A)** CT scan; **(B)** MRI scan; **(C)** any angiography.

In 15 (12.9%) of cases, initial diagnostic CT was not performed, while primary MRI scan was performed instead. In those undergoing primary CT scan without contrast, in only 16.0% of cases, CT was able to depict possible underlying cause of ICH. MRI scans revealed underlying structural causes, such as bleeding cavernomas in 31 cases (26.7%) and arteriovenous malformations (AVM) in 12 cases (10.3%). MRI was able to depict absent signal void in venous sinuses, revealing CVT in nine cases (7.8%). In 55 (47.4%) cases, MRI finding was non-specific. Any angiographic study was performed in 79 (68.1%) of patients, of which 52 (44.8%) studies were normal. CTA or MRA were performed in 44 (37.9%) and 49(42.2%), respectively, which confirmed the diagnoses of vasculitis in two cases, and one case was revealed to be moyamoya disease. DSA revealed more AVM cases (*n* = 15; 12.9%) compared with MRI. Neuroimaging performed in the study population and radiological diagnoses are shown in Figure 3.

### Sensitivity and Specificity of CT, MRI, and Angiography for Detecting Structural/Local Causes

We compared the sensitivity and specificity of CT, MRI, and angiography for the detection of different structural/local causes for all patients and also stratified to age groups and time interval between ICH incidence and MRI (Table 3). CT showed not only low sensitivity (26.5%) in the detection of structural/local causes but also high specificity (86.3%) compared with MRI. MRI was found to be more sensitive (90.0%) in detecting structural/local causes compared with any angiographic imaging (55.5%) including CTA, MRA, or DSA. However, MRI was found to be less specific (87.3%) for structural/local causes compared with angiography (97.4%).

**Table 3:** Sensitivity and specificity of CT, MRI, and angiographic imaging in detecting structural/local causes underlying intracerebral hemorrhage.

	**CT**		**MRI**		**Angiography**	
**All**	**Value**	**95% CI**	**Value**	**95% CI**	**Value**	**95% CI**
Sensitivity (%)	26.5	15.0–41.1	90.0	79.5–96.2	55.6	40.0–70.4
Specificity (%)	86.3	73.7–94.3	87.3	75.5–94.7	97.4	86.5–99.9
PPV (%)	65.0	44.7–81.0	88.5	79.4–93.9	96.2	78.0–99.4
NPV (%)	55.0	50.0–60.0	88.9	78.8–94.5	65.5	57.7–72.6
Accuracy (%)	57.0	46.7–66.9	88.7	81.5–93.8	75.0	64.4–83.8
**AGE <39 YEARS**						
Sensitivity (%)	26.7	12.3–45.9	89.5	75.2–97.1	57.1	37.2–75.5
Specificity (%)	79.0	54.4–94.0	85.0	62.1–96.8	93.8	69.8–99.8
PPV (%)	66.7	41.1–85.2	91.9	79.9–97.0	94.1	70.0–99.1
NPV (%)	40.5	33.2–48.4	81.0	62.3–91.6	55.6	44.5–66.1
Accuracy (%)	46.9	32.5–61.7	87.9	76.7–95.0	70.5	54.8–83.2
**AGE ≥39 YEARS**						
Sensitivity (%)	26.3	9.2–51.2	90.9	70.8–98.9	52.9	27.8–77.0
Specificity (%)	90.6	75.0–98.0	88.6	73.3–96.8	100.0	85.2–100.0
PPV (%)	62.5	30.9–86.1	83.3	66.3–92.7	100.0	–
NPV (%)	67.4	60.8–73.5	93.9	80.4–98.3	74.2	63.5–82.6
Accuracy (%)	66.7	52.1–79.2	89.5	78.5–96.0	80.0	64.4–91.0
**MRI WITHIN ≤2 WEEKS FROM ICH ONSET**						
Sensitivity (%)	25.0	12.7–41.2	90.2	78.6–96.7	62.2	44.8–77.5
Specificity (%)	82.8	64.2–94.2	86.7	69.3–96.2	96.0	79.7–99.9
PPV (%)	66.7	43.3–84.0	92.0	82.1–96.6	95.8	76.8–99.4
NPV (%)	44.4	38.5–50.5	83.9	69.1–92.4	63.2	53.0–72.3
Accuracy (%)	49.3	37.0–61.6	88.9	80.0–94.8	75.8	63.3–85.8
**MRI >2 WEEKS FROM ICH ONSET**						
Sensitivity (%)	33.3	7.5–70.1	88.9	51.8–99.7	25.0	3.2–65.1
Specificity (%)	90.9	70.8–98.9	88.0	68.8–97.5	100.0	76.8–100.0
PPV (%)	60.0	23.0–88.3	72.7	47.4–88.8	100.0	–
NPV (%)	76.9	67.3–84.4	95.7	77.5–99.3	70.0	61.0–77.7
Accuracy (%)	74.2	55.4–88.1	88.2	72.6–96.7	72.7	49.8–89.3

*CI, confidence interval; PPV, positive predictive value; NPV, negative predictive value*.

Sensitivity and specificity analysis of different imaging modalities did not change markedly between age groups; sensitivity of MRI to detect structural/local causes in the younger age group was 89.5 vs. 90.9% in the older age group. The time interval between ICH onset and imaging acquisition also did not affect the sensitivity and specificity of CT and MRI. However, we noticed marked decrease in angiographic imaging sensitivity when performed >2 weeks after ICH onset compared with early (<2 weeks) imaging.

## Discussion

Our study demonstrated that MRI had higher sensitivity for detecting structural and local causes compared with CT and angiography, whereas angiographic imaging was highly specific for structural abnormalities. Structural/local causes were the leading underlying causes of our study participants, and of these, bleeding cavernous malformations were the most common followed by arteriovenous malformations. We found also that lobar location of ICH was more prevalent in the younger age group, while deep location (ganglionic or thalamic) was more prevalent in the older age group.

Studies investigating the role of MRI in younger ICH patients are scanty. One study reported MRI sensitivity as high as 93% and specificity of 100%; however, patients included in that study were of older age group and MRI was obtained in only 23% of all ICH patients (24). Prospective studies are warranted to explore the optimal set of MRI sequences and timing of imaging in the etiological work-up of young ICH patients.

In 70.7% of cases, CT imaging suggested non-specific findings and further imaging needed to determine the underlying cause. Performing MRI scans, either primarily or secondarily, revealed structural causes, with ability to depict cavernomas and AVM more accurately, especially in cases of multiple cavernomas including non-bleeding cavernomas. Other rare causes like vasculitis and CVT could be identified, which needed further angiographic imaging to confirm diagnosis. In our study, the utility of primary MRI in young ICH patients revealed more frequently structural abnormalities as underlying cause compared with other studies. Structural causes were leading causes of young adult ICH in 50.0% of all cases, with cavernomas being the most common (23.3%) of these. Other structural causes included AVM, CVT, moyamoya disease, and brain tumors. The second most common causes were systemic diseases (16.4%) followed by hypertension (15.5%). Among the 13 studies included in the systematic review of ICH in young adults (1), 7 studies reported hypertension as leading cause of ICH in young patients (ranging from 25.0 to 79.2%), while in 5 studies, AVMs were the leading underlying cause (ranging from 10.8 to 29.1%). One small American study reported aneurysms as the leading cause of all study patients (*n* = 46; 45.6%) (12).

ICH imaging characterization aids by detecting possible underlying causes and guide treatment. In our study, we characterized location, side, shape, and volume of ICH and whether microbleeds or any other adjunct features or complications like IVH, hydrocephalus, or both are present. One Indian study have found deep-seated location of ICH in young patients was more prevalent (71.3%) than lobar location (7.2%) (16). In contrast, we found that location of bleeding was significantly different between age groups in our study, with lobar location being more prevalent in the younger age group. Most likely, this observation occurred due to the higher frequency of cavernomas, AVMs, and CVTs among younger patients. In the few studies including MRI in their methodology, ICH was characterized based on location only (3, 9, 16). We noted, for example, that lobar location in the younger age group indicates probably underlying vascular malformations, while deeply lenticulostriate or thalamic location was more prevalent in the older age group due to the higher likelihood of hypertensive small-vessel disease. Among structural causes, location of ICH was mainly lobar, compared with non-structural causes, where ICH was mainly deep in location. Shape of ICH in lobar location was mostly irregular, while deeper hematomas were more regular in shape. There was no side preference for ICH. In our study population, in those whom MRI sequences included T2^*^ or SWI (75.0%), the presence of CMBs was infrequent (10.0%). Microbleeds were significantly more prevalent in non-structural causes, with only one case of structural etiology reporting the presence of microbleeds (Table 4).

**Table 4:** Comparison of intracerebral hemorrhage imaging features between structural/local and non-structural causes (other/undetermined).

	**Structural/local causes (*n* = 58)**	**Other/undetermined causes (*n* = 58)**	***P-*value**
**LOCATION**			0.010
Lobar	34 (58.6%)	22 (37.9%)	
Basal ganglia/thalamus	7 (12.1%)	20 (34.5%)	
Brain stem	6 (10.3%)	9 (15.5%)	
Cerebellum	0 (0.0%)	2 (3.4%)	
Intraventricular	8 (13.8%)	2 (3.4%)	
Mixed	3 (5.2%)	3 (5.2%)	
**SITE**			0.230
Right	20 (34.5%)	20 (34.5%)	
Left	26 (44.8%)	28 (48.3%)	
Bilateral	3 (5.2%)	4 (6.9%)	
Midline	1 (1.7%)	4 (6.9%)	
Isolated IVH	8 (13.8%)	2 (3.4%)	
**SHAPE**			0.115
Regular	16 (27.6%)	10 (17.2%)	
Irregular	34 (58.6%)	44 (75.9%)	
Mixed/multiple bleedings	0 (0.0%)	1 (1.7%)	
Not applicable	8 (13.8%)	3 (5.2%)	
**MICROBLEEDS**			0.008
No	39 (67.2%)	38 (65.5%)	
Yes	1 (1.7%)	10 (17.2%)	
Not assessable	18 (31.0%)	10 (17.2%)	
**COMPLICATIONS**			0.381
None	36 (62.1%)	33 (56.9%)	
IVH/SAH	9 (15.5%)	14 (24.1%)	
Hydrocephalus	4 (6.9%)	1 (1.7%)	
Both	9 (15.5%)	10 (17.2%)	

*IVH, intraventricular hemorrhage; SAH, subarachnoid hemorrhage*.

The volume of ICH is of clinical importance as it correlates with mortality and functional outcome (3). The median volume of ICH in our study was 7 ml. All our study patients (with or without initial CT) underwent brain MRI. MRI showed higher detection rates for vascular malformations (40.4%) with ability to specify the type of malformation. CT only is neither sensitive nor specific for detection of cavernomas. Therefore, MRI is the diagnostic modality of choice in young adult patients, particularly when vascular etiology is clinically suspicious (25). The advantage of using gradient echo (T2^*^) sequences and susceptibility-weighted sequences renders MRI more sensitive for detection of chronic microangiopathies (19, 20). Only a few studies reported the usage of MRI as secondary imaging modality in the diagnostic work-up of ICH in young patients (3, 5, 6). A recent study which did not report imaging modalities' proportions used in the study, showed structural causes representing only 4.2% of ICH etiology (16). Another study, despite reporting that no MR imaging was performed, showed notably high detection of structural causes (AVM, 29.1%; aneurysms, 9.7%; moyamoya, 1.4%); however, this might be explained by the extensive (84.7%) angiographic imaging (13).

DSA is so far the gold standard imaging technique for detection of AVMs (26, 27). However, we noticed that MRI is more sensitive in detection of other structural causes such as cavernomas and tumors. Angiographic imaging including CTA, MRA, and DSA was more specific for structural causes, such as vascular malformations. A prospective diagnostic study including 298 patients showed little added value of MRI/MRA combined to early CT angiography, compared with CT angiography only in detection of macrovascular causes. This is probably explained by the older study population (mean, 53.0 years) compared with our study (mean, 36.8 years) and hence the lower incidence of structural etiologies (28). Furthermore, MRI is superior in detection of chronic microhemorrhages using the advantage of T2^*^ and SWI techniques. Due to the more invasive nature of catheter imaging and its possible complications (4%) (23, 29, 30), MRI remains the safer imaging technique in the diagnostic process of ICH in young patients.

The optimal timing of MRI imaging in diagnostic work-up of ICH in the young has not been prospectively studied. In our study, sensitivity and specificity of imaging modalities did not markedly change when comparing different age groups. The time interval between ICH onset and imaging acquisition also did not remarkably affect the sensitivity and specificity of CT and MRI. However, angiographic imaging sensitivity noticeably decreased when performed >2 weeks from onset of ICH, compared with early performed imaging.

The main limitations of our study include its retrospective nature and the relatively modest sample size. A standardized MRI protocol could not be applied to all patients. We further had to limit the sample of CT- and MRI-imaged patients, as in 15 patients, initial emergent CT was not performed, either due to young age or other contraindications to radiations like pregnancy. Also, our database consists of a relative old dataset. Since then, imaging strategies may have changed or even have been improved (for example, more advanced CT techniques such as 4D-CTA have been introduced). However, to the best of our knowledge, our study represents one of the largest series of MR-imaged young ICH patients and strongly supports the wider use of MRI in the diagnostic work-up of this patient group.

## Data Availability Statement

The raw data supporting the conclusions of this article will be made available by the authors, without undue reservation.

## Ethics Statement

Ethical review and approval was not required for the study on human participants in accordance with the local legislation and institutional requirements. Written informed consent for participation was not required for this study in accordance with the national legislation and the institutional requirements.

## Author Contributions

ME, JP, and JM outlined the study design. JP and R-JK collected data. JM and ME analyzed imaging data. ME and JP organized data and performed statistical data analysis. ME drafted and finalized the manuscript. JP, R-JK, TT, and JM reviewed and commented on the manuscript. All authors revised the final submitted version of the manuscript. All authors contributed to the article and approved the submitted version.

## Conflict of Interest

JM Lecture honoraria Santen. The remaining authors declare that the research was conducted in the absence of any commercial or financial relationships that could be construed as a potential conflict of interest. The reviewer FS declared a past co-authorship with one of the authors JP to the handling editor.
